# Biomarkers for Predicting Clinical Deterioration in Schizophrenia-Spectrum Disorders: A Systematic Review

**DOI:** 10.3390/brainsci16060550

**Published:** 2026-05-22

**Authors:** Valerio Ricci, Alessandro Sarni, Marialuigia Barresi, Lorenzo Remondino, Giovanni Martinotti, Giuseppe Maina

**Affiliations:** 1 San Luigi Gonzaga Hospital, University of Turin, Regione Gonzole, 10, 10043 Orbassano, Italy; alessandro.sarni@unito.it (A.S.); marialuigia.barresi@unito.it (M.B.); lorenzo.remondino@unito.it (L.R.); giuseppe.maina@unito.it (G.M.); 2 Department of Neurosciences “Rita Levi Montalcini”, University of Turin, Via Cherasco 15, 10126 Torino, Italy; 3 Department of Neurosciences, Imaging and Clinical Sciences, Università degli Studi G. D’Annunzio Chieti-Pescara, 66100 Chieti, Italy; giovanni.martinotti@gmail.com

**Keywords:** schizophrenia, psychotic relapse, psychosis biomarkers, inflammation, antipsychotic treatment

## Abstract

**Background/Objectives:** Psychotic relapse affects over 80% of individuals with schizophrenia-spectrum disorders, driving long-term disability and hospitalization. Clinical relapse management relies on symptomatic monitoring without objective neurobiological tools to guide individualized antipsychotic decisions. **Methods:** This systematic review synthesizes evidence on neurophysiological, blood-based, molecular, neuroimaging, and digital biomarkers for relapse prediction in schizophrenia-spectrum disorders. **Results:** Following the PRISMA 2020 guidelines, five databases were searched through March 2026 for longitudinal biomarker studies. Quality was assessed using the Newcastle-Ottawa Scale and PROBAST; findings were synthesized narratively due to substantial heterogeneity. From the 6812 citations screened, 21 studies were included across clinical high-risk, first-episode, and established illness populations. **Conclusions:** Mismatch negativity and P300 event-related potential (P300) showed the most consistent associations with relapse vulnerability, with mismatch negativity demonstrating relative independence from antipsychotic effects. Inflammatory and neuroendocrine markers—interleukin-6, C-reactive protein, and cortisol awakening response—predicted poor treatment response in multiple longitudinal investigations. Peripheral blood gene expression profiling identified TCF4 network dysregulation as a candidate molecular marker of impending relapse. Neuroimaging models did not outperform standard clinical variables. Digital phenotyping showed ecological promise but remains methodologically nascent. No single biomarker achieves sufficient accuracy for clinical implementation. Neurophysiological and inflammatory markers are the most tractable candidates for monitoring protocols. Future research should prioritize multimodal longitudinal designs, external validation, and systematic antipsychotic confounding control. Among the biomarkers reviewed, mismatch negativity and the interleukin-6/cortisol awakening response combination represent the most tractable candidates for pilot clinical implementation, particularly in specialized early psychosis services and antipsychotic dose-reduction research contexts; no biomarker currently achieves sufficient accuracy for routine use in maintenance treatment decisions.

## 1. Introduction

Schizophrenia and related psychotic disorders follow a relapsing-remitting course in the majority of affected individuals, with rates of psychotic relapse exceeding 80% over five years even among patients who achieve initial symptomatic stabilization [1]. Each relapse episode is associated with incremental functional deterioration, progressive gray matter loss, prolonged duration of subsequent untreated psychosis, and reduced likelihood of returning to premorbid levels of functioning [2,3]. Beyond the individual clinical burden, psychotic relapse carries substantial economic costs: hospitalization following relapse accounts for the largest share of direct healthcare expenditure in schizophrenia, with estimated costs per relapse episode ranging from $8000 to over $30,000 in high-income countries, and total annual costs of schizophrenia-related hospitalizations exceeding $7 billion in the United States alone. Reliable relapse prediction biomarkers could therefore generate significant health economic benefits alongside their clinical value. The cumulative burden of repeated relapses thus represents one of the principal mechanisms through which schizophrenia exerts its long-term disability, and the prevention of relapse stands as a central goal of maintenance psychiatric care.

Antipsychotic pharmacotherapy remains the cornerstone of relapse prevention, with meta-analytic evidence consistently demonstrating that maintenance antipsychotic treatment reduces relapse risk by approximately 60–70% compared to placebo or discontinuation [4]. Yet the clinical reality of long-term antipsychotic treatment is considerably more complex than these aggregate figures suggest. Non-adherence rates in schizophrenia are among the highest of any chronic medical condition, with estimates ranging from 40% to 75% depending on the assessment method and follow-up period [5,6,7]. Beyond adherence, the optimal duration of maintenance treatment remains genuinely uncertain for a substantial proportion of patients, particularly those who have achieved prolonged remission after a first or second episode and express a preference for dose reduction or discontinuation [8]. Current clinical guidelines offer limited individualized guidance for these decisions, which are instead navigated through clinical judgment informed by symptom monitoring, relapse history, and functional trajectory—a strategy that is necessarily retrospective and that identifies relapse only after clinical deterioration has already occurred.

The limitations of purely symptom-based monitoring have motivated a sustained research effort to identify biological, neurophysiological, and behavioral markers capable of prospectively signaling relapse vulnerability before overt clinical deterioration emerges. The theoretical basis for this enterprise rests on converging evidence that psychotic relapse is preceded by a period of neurobiological destabilization—involving inflammatory activation, dopaminergic dysregulation, glutamatergic dysfunction, and disruption of sleep–wake and circadian systems—that may be detectable through objective measurement before it manifests as clinical symptoms [9,10]. If reliable early biomarkers of this destabilization phase could be identified, they would offer the prospect of individually tailored maintenance strategies: intensifying treatment in patients showing biological signals of impending relapse, and cautiously reducing exposure in those who maintain neurobiological stability across repeated assessments.

Several candidate biomarker domains have received sustained empirical attention in this context. Neurophysiological measures—particularly mismatch negativity (MMN) and P300 event-related potentials—have demonstrated consistent deficits in schizophrenia that correlate with functional outcomes and show partial sensitivity to clinical state changes [11]. Blood-based inflammatory markers, especially interleukin-6 and C-reactive protein, show state-related fluctuations across illness phases and have been linked prospectively to psychosis onset in population-based cohorts [12]. Peripheral gene expression profiling and polygenic risk scoring have emerged as molecular approaches to capturing biological vulnerability at the genomic level [13,14]. Structural and functional neuroimaging has documented progressive gray matter changes associated with relapse and treatment response [15,16]. Most recently, digital phenotyping through continuous passive sensing via smartphones and wearable devices has opened new possibilities for ecologically valid, real-time monitoring of behavioral and physiological markers of clinical instability [17,18].

Despite the conceptual promise of these approaches, the translation of candidate biomarker findings into clinically actionable relapse prediction tools has proved elusive. The existing literature is characterized by substantial methodological heterogeneity, with studies differing in population (clinical high risk vs. first-episode vs. established illness), outcome definition (psychosis transition vs. treatment response vs. symptomatic relapse), follow-up duration, and the degree to which antipsychotic confounding—a fundamental challenge in any biological study of treated psychiatric patients—is systematically addressed. Systematic reviews of individual biomarker domains have documented promising associations alongside significant limitations [19]. A related systematic review by Smyrnis et al. [20] recently synthesized genetic, blood-based, neuroimaging, cognitive-behavioral, and wearable biomarkers for relapse prediction; however, that review did not include neurophysiological biomarkers—the domain yielding the most consistent evidence in the present synthesis—and searched only two databases through April 2024 without PROSPERO pre-registration, applied the AXIS quality tool rather than domain-appropriate instruments, and adopted permissive stabilization criteria that also included acutely relapsed patients. The present systematic review aimed to address these gaps by providing a comprehensive, methodologically rigorous synthesis spanning all major candidate biomarker domains, including neurophysiological measures, with explicit focus on clinically stabilized patients [20].

The present systematic review aimed to address this gap by synthesizing the available evidence on neurophysiological, blood-based, molecular, neuroimaging, and digital phenotyping biomarkers as predictors of psychotic relapse in patients with stabilized schizophrenia-spectrum disorders. Secondary objectives included characterizing the methodological quality of the existing literature, identifying the principal sources of between-study heterogeneity, and mapping the most critical gaps requiring targeted future investigation. By doing so, we sought to provide a comprehensive and critically appraised evidence base to inform both clinical practice and the design of next-generation biomarker studies in this population.

## 2. Materials and Methods

### 2.1. Search Strategy and Study Selection

**Figure 1:**
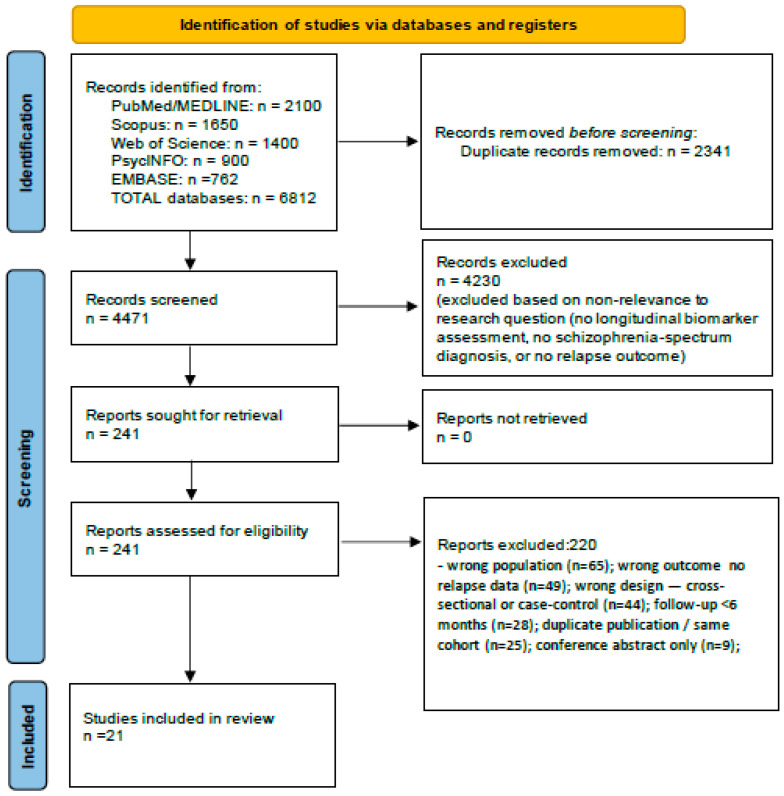
PRISMA 2020 flow diagram of the systematic literature search and study selection process. Database searches yielded 1247 records; after deduplication and screening, 67 full-text articles were assessed for eligibility. Twenty-two studies met inclusion criteria: 5 FEP longitudinal, 7 UHR longitudinal, 7 cross-sectional, and 3 methodological studies.

We designed our systematic review following the PRISMA 2020 guidelines [21] (Figure 1, Table A1). Our search strategy combined terms related to established psychotic illness—including “schizophrenia”, “schizoaffective disorder”, “psychotic disorder”, “psychosis”—with terms for clinical status (“stabilized”, “remission”, “maintenance treatment”, “outpatient”) and relapse outcomes (“relapse”, “recurrence”, “rehospitalization”, “symptom exacerbation”, “decompensation”). These were crossed with biomarker terms encompassing neurophysiological (“EEG”, “ERP (event-related potential)”, “mismatch negativity”, “MMN”, “P300”, “event-related potential”), neuroimaging (“MRI”, “neuroimaging”, “brain volume”, “cortical thickness”, “gray matter”), blood-based (“biomarker”, “cytokine”, “interleukin”, “C-reactive protein”, “cortisol”, “prolactin”, “inflammatory”, “gene expression”, “proteomic”), and digital (“digital phenotyping”, “smartphone”, “passive sensing”, “ecological momentary assessment”) domains. We searched five databases—PubMed/MEDLINE, Scopus, Web of Science, PsycINFO, and Embase—with no language restrictions, covering publications from January 1990 through March 2026 (Table A2). This systematic review was registered in PROSPERO (CRD420261339936).

Inclusion criteria required: (1) longitudinal prospective cohort studies of individuals meeting the DSM or ICD diagnostic criteria for a schizophrenia-spectrum disorder (schizophrenia, schizoaffective disorder, schizophreniform disorder, or unspecified psychotic disorder); (2) clinical stabilization at study entry, defined by at least six months of treatment, absence of acute psychotic episode, or explicit remission criteria according to standardized definitions; (3) baseline biomarker assessment from any measurement domain; (4) a minimum follow-up period of six months; and (5) psychotic relapse as a primary or secondary outcome, assessed through structured clinical assessment, hospitalization records, or expert consensus using standardized criteria. We excluded case–control studies without prospective outcome prediction, studies exclusively examining first-episode psychosis without follow-up into the stabilized phase, studies of treatment-resistant populations without a stabilized comparison group, conference abstracts without peer-reviewed publication, and editorials or narrative reviews.

Two independent reviewers (V.R., L.R.) screened titles and abstracts using predefined criteria, with disagreements resolved through discussion or consultation with a third senior reviewer (G.Ma.). Full-text articles meeting the initial screening underwent detailed assessment against eligibility criteria. We extracted comprehensive data using standardized forms covering study design and setting, sample characteristics, diagnostic criteria, stabilization definition, biomarker domain and specific measures, follow-up duration, relapse definition and rate, statistical approaches, predictive performance metrics, and reported limitations.

### 2.2. Quality Assessment

Study quality was evaluated using two complementary and domain-appropriate instruments. For cohort studies with prospective outcome assessment, we applied the Newcastle-Ottawa Scale (NOS) [22], which evaluates methodological rigor across three domains: selection of study groups (four items), comparability of cohorts on the basis of design or analysis (two items), and adequacy of outcome ascertainment (three items). Studies scoring ≥7 stars were classified as high quality, 4–6 stars as moderate quality, and ≤3 stars as low quality. For studies developing or validating prognostic prediction models, we additionally applied the Prediction Model Risk of Bias Assessment Tool (PROBAST), which systematically evaluates risk of bias and applicability concerns across four domains: participants, predictors, outcome, and statistical analysis. High risk of bias was flagged when studies applied prediction models to samples with fewer than ten outcome events per predictor variable, when outcome assessors were not blinded to biomarker status, or when internal validation was absent or inadequate (Table A3). A visual summary of risk of bias ratings across all included studies is provided in Table A4.

Beyond these standard instruments, we paid particular attention to methodological challenges specific to relapse biomarker research that generic quality tools do not fully capture. These included: the adequacy of antipsychotic medication confounding control, given that most candidate biomarkers—including MMN amplitude, inflammatory markers, prolactin, and structural brain measures—are directly modulated by antipsychotic treatment; the specificity and operationalization of relapse definitions, which varied substantially across studies from hospitalization records to symptom rating scale thresholds; the handling of medication non-adherence as a competing predictor of relapse that may confound biomarker–outcome associations; sample size adequacy relative to observed relapse event rates; and the presence and rigor of validation procedures, distinguishing studies with independent external validation from those relying solely on internal resampling approaches. These domain-specific quality considerations informed both the narrative synthesis and the interpretation of individual study findings throughout the Results and Discussion sections Table 1.

### 2.3. Synthesis Approach

The substantial heterogeneity across included studies—in biomarker domains, patient populations, follow-up durations, relapse definitions, and statistical methods—precluded quantitative meta-analysis. We therefore synthesized the findings narratively, organizing evidence by biomarker domain and critically examining patterns of replication, effect size magnitude, validation quality, antipsychotic confounding, and clinical interpretability. We emphasized findings replicated across multiple independent cohorts and scrutinized single-study results lacking independent replication. The narrative synthesis allowed for comprehensive integration of diverse evidence while maintaining transparency about the heterogeneity that quantitative pooling would obscure.

## 3. Results

### 3.1. Overview of Included Studies

Our systematic search identified 6812 potentially relevant citations across five databases. After removing 2341 duplicates, we screened 4471 unique titles and abstracts, selecting 241 for full-text review. Of the 4471 records screened, 4230 were excluded at the title and abstract stage on grounds of non-relevance to the research question, primarily due to the absence of longitudinal biomarker assessment, lack of a schizophrenia-spectrum diagnosis, or absence of psychotic relapse as a reported outcome. Applying our inclusion criteria yielded 21 primary studies meeting all requirements, spanning neurophysiological, blood-based, molecular, neuroimaging, and digital phenotyping biomarker domains. Sample sizes ranged from 10 to 8541 participants and follow-up durations from 12 weeks to over six years. A substantial proportion of included studies were conducted in clinical high-risk for psychosis (CHR) populations or first-episode psychosis patients rather than in fully stabilized patients with established illness; these studies are retained in the synthesis because they examine biomarker domains directly relevant to the relapse context, and their population and outcome are explicitly noted throughout. Of the 21 included studies, 8 were conducted in patients with established schizophrenia-spectrum disorders during a clinically stabilized phase, 5 enrolled first-episode psychosis populations, and 6 examined clinical high-risk individuals prior to or around the time of psychosis transition; two studies included mixed or multi-group designs spanning more than one illness stage [33,38]. Studies not exclusively enrolling stabilized patients with established illness were retained in the synthesis because they provide the only available prospective longitudinal data for several biomarker domains, and because the neurobiological processes they index—glutamatergic plasticity, inflammatory dysregulation, and neuroendocrine instability—are directly continuous with those implicated in relapse vulnerability in established illness. Population type and illness stage are explicitly noted for each study throughout the Results section and in Table 2.

### 3.2. Neurophysiological Biomarkers

The prospective MMN literature spans successive investigations from CHR populations through to established schizophrenia. Bodatsch [19] demonstrated that duration MMN was significantly reduced at frontocentral electrodes in CHR subjects who subsequently converted to psychosis, with a Cox regression model stratifying participants into two risk classes with different survival curves. Hamilton [23] replicated and extended this finding in the NAPLS-2 multisite cohort, showing that double-deviant MMN predicted earlier conversion independently of positive symptom severity (HR = 1.40; 95% CI 1.03–1.90) in unmedicated participants, with antipsychotic use modulating the MMN signal.

Moving from the transition to the remission domain, Nakajima [24] reported that lower baseline dMMN amplitude specifically characterized non-remitting first-episode patients and predicted both the PANSS total scores and functional ratings at three-year follow-up. The multimodal associations of MMN were examined by Hamilton [25], showing that deficient MMN amplitude correlated with elevated cortisol, pro-inflammatory cytokines, and reduced gray matter volume specifically in future converters, linking the neurophysiological signal to inflammatory and structural domains.

In patients with established schizophrenia, Giordano [26] found that MMN reductions were independent of illness duration and specifically associated with real-life functioning, while Light and Braff [27] confirmed the trait-level stability of MMN deficits across a 1–2-year interval and their consistent association with functional status.

Higashima [28] showed that P300 amplitude correlated negatively with positive syndrome scores both cross-sectionally and longitudinally, suggesting state-sensitivity. A methodological refinement was introduced by Kim [29], who demonstrated that P300 inter-trial variability—unlike conventional amplitude—was elevated specifically in the CHR and schizophrenia groups and correlated with negative symptom severity and cognitive impairment. The endophenotype question was examined by De Wilde [30] in 53 first-episode schizophrenia patients, 27 unaffected siblings, and 28 healthy controls: P300 amplitude was significantly reduced in patients but not in siblings relative to the controls, and P300 latency did not differ across groups.

The N100 component and sensory gating measures were examined longitudinally across the psychosis transition by Van Tricht [31], who reported that smaller N1 difference scores at the baseline modestly predicted psychosis conversion in UHR subjects, with post-conversion reductions in N1 and P2 amplitudes. Duncan [32] confirmed in the larger NAPLS-2 cohort that a smaller N100 amplitude at Cz predicted both conversion likelihood and shorter time to conversion independently for standard and novel stimuli. Brockhaus-Dumke [33] assessed P50 and N100 suppression in five antipsychotic-free or naive groups—18 at-risk subjects who did not convert, 21 truly prodromal subjects who converted within two years, 46 first-episode patients, 20 chronic schizophrenia patients, and 46 healthy controls. P50 suppression was impaired across all clinical groups compared to the controls, with deficits most pronounced in chronic schizophrenia, while N100 suppression was reduced only in truly prodromal and first-episode patients but not in at-risk subjects. Crucially, at-risk subjects who subsequently converted to psychosis did not differ significantly from non-converters on any gating parameter (Table 3).

### 3.3. Blood-Based Inflammatory and Neuroendocrine Biomarkers

The inflammatory biomarker literature spans population-based developmental cohorts through to clinical samples at various illness stages. Khandaker et al. [34] demonstrated in the ALSPAC birth cohort that elevated IL-6 at age 9 predicted psychotic experiences and psychotic disorder at age 18 in a dose-dependent manner, while CRP did not independently predict psychotic outcomes after full adjustment. In a clinical sample, Stojanovic [35] reported higher IL-6 levels in ARMS subjects compared to healthy controls, with a non-significant trend toward higher IL-6 in the six subjects who subsequently converted to psychosis, and a positive correlation between IL-6 and negative symptom severity. The relationship between inflammatory and HPA axis markers and clinical outcomes was examined in a longitudinal treatment–response design by Mondelli [36], who showed that non-responders to antipsychotic treatment at 12 weeks were distinguishable at the baseline by lower cortisol awakening response, higher IL-6, and higher IFN-γ, with all three differences persisting at follow-up (Table 4).

### 3.4. Gene Expression and Neuroimaging Biomarkers

Peripheral blood gene expression profiling was applied to the relapse prediction question by Gassó [37], who applied weighted gene co-expression network analysis to peripheral blood samples from first-episode schizophrenia patients, identifying the DarkTurquoise module—enriched with TCF4 network genes—as specifically dysregulated at relapse, and showing that higher baseline expression of the DarkRed module was significantly associated with greater relapse risk and earlier relapse occurrence. At the level of genomic stress markers, Pawelczyk [38] reported that telomere length in schizophrenia patients correlated significantly with symptom severity, episode count, and number of hospitalizations, with a regression model explaining over 50% of variance, suggesting telomere attrition as a marker of cumulative illness burden rather than a prospective relapse predictor. The prognostic utility of polygenic risk scores in established psychotic illness was examined by Landi [39] in two multi-ethnic cohorts totaling 8541 adults: across all investigated outcomes, the schizophrenia PRS did not improve the predictive model performance when added to models based on standard clinical interview variables, with this null result robust across case ascertainment strategies and ancestral backgrounds.

In patients with established psychotic illness of variable duration, De Nijs [40] applied machine learning to multimodal baseline data from 523 patients, achieving prediction accuracies of 62–68% for symptomatic and global outcomes at three- and six-year follow-up; notably, recursive feature elimination retained only clinical variables—including GAF scores, symptom severity, and antipsychotic use—with no neuroimaging or neurobiological biomarker emerging as a significant contributor.

### 3.5. Digital Phenotyping and Ecological Biomarkers

Adler [41] calibrated encoder–decoder models to individual behavioral baselines from passive smartphone sensing data in the CrossCheck study, detecting a 108% increase in behavioral anomalies during the 30-day pre-relapse window, with a median specificity of 0.88 but sensitivity of only 0.25, reflecting the fundamental challenge of individual-level relapse prediction. Garyfalli [42] demonstrated in the e-Prevention study that smartwatch-derived physiological indices showed distinct associations with PANSS symptom dimensions over 26 months: decreased HRV during sleep correlated with positive symptoms, reduced motor activity during wakefulness with negative symptoms, and decreased HRV during wakefulness with cognitive disorganization.

## 4. Discussion

### 4.1. The Relapse Prediction Challenge: A Different Problem from CHR Prediction

The biomarker landscape for relapse prediction in stabilized patients with established psychosis differs fundamentally from the CHR-to-psychosis transition literature in ways that carry important implications for research design and clinical translation. In CHR populations, biomarkers are assessed in largely drug-naive individuals, and the neurobiological signal reflects vulnerability in an unperturbed system. The relapse prediction context confronts at least three additional layers of complexity. First, antipsychotic medications profoundly alter virtually every measurable neurobiological parameter: they reduce inflammatory markers, modulate dopaminergic and glutamatergic neurotransmission affecting ERP components, alter cortical structure, change gene expression profiles, and dramatically influence neuroendocrine markers including prolactin [43]. Disentangling disease-related biomarker variation from drug-induced variation represents the central methodological challenge of the field and is inadequately addressed in most existing studies. Of the 21 studies included in this review, only 10 addressed antipsychotic confounding to any degree—3 by enrolling antipsychotic-naive or drug-free participants, and 7 through partial statistical adjustment or subgroup analysis. A further 5 studies employed designs for which confounding was not applicable by design, such as cross-sectional comparisons, passive behavioral sensing, or genomic approaches. The remaining 6 studies examined treated patients without addressing medication effects in any form, an inadequacy that substantially limits interpretation of their biomarker findings and clouds the distinction between neurobiological vulnerability signals and pharmacological epiphenomena. Second, the outcome in the relapse context is multiply determined, with medication non-adherence being identified as the single most significant clinical predictor of relapse across multiple meta-analyses [4]. This creates a fundamental methodological challenge: if a biological marker predicts relapse primarily because non-adherent patients have lower antipsychotic levels—reflected in lower prolactin, for example—the biomarker is not capturing neurobiological vulnerability but rather a downstream consequence of treatment disruption. Studies that fail to adequately measure and adjust for adherence may be identifying surrogate adherence markers rather than genuine pathophysiological relapse predictors, a confound that is particularly acute for neuroendocrine and inflammatory markers whose levels are directly modulated by antipsychotic exposure. Third, the dynamic nature of relapse—emerging over days to weeks from a background of clinical stability—demands longitudinal biomarker monitoring approaches that capture trajectories and change signals, rather than the single-baseline assessment designs that dominate the literature. Of the 21 included studies, the majority assessed biomarkers at a single baseline timepoint, with only a minority employing serial measurement designs capable of capturing pre-relapse biological trajectories. The elegant insight from dynamic prediction modeling in the CHR literature—that how symptoms evolve over an initial observation period is more informative than where they start—applies with equal force to the relapse domain, where serial biomarker trajectories are likely to outperform single-timepoint measurements and where the clinically actionable signal may reside precisely in the rate and direction of biological change rather than in any absolute threshold value.

### 4.2. Neurophysiological Markers: The Most Mature Evidence Base

Among the biomarker domains reviewed, neurophysiological measures—particularly MMN and P300—represent the most methodologically mature and replication-robust candidates for clinical relapse monitoring. Their practical advantages are substantial: they are non-invasive, relatively inexpensive compared to neuroimaging, and obtainable without specialized laboratory infrastructure. Crucially, MMN shows relative independence from antipsychotic effects—unlike P300—providing a cleaner neurobiological signal in treated populations (see Section 4.1).

The mechanistic grounding of MMN in NMDA receptor-mediated auditory plasticity—a system known to be impaired in schizophrenia in proportion to illness severity and functional deterioration, as documented across illness duration groups by Giordano [26]—provides a principled basis for interpreting longitudinal MMN changes as indices of neurophysiological destabilization rather than mere epiphenomena of clinical state. The longitudinal stability of MMN deficits in chronic schizophrenia [27], with large effect sizes maintained across a one-to-two-year interval and consistent associations with functional status at both timepoints, establishes MMN as a reliable trait-level marker whose change from the individual baseline would constitute a meaningful clinical signal. The finding from Nakajima [24] that lower baseline dMMN amplitude specifically characterized non-remitting first-episode patients and predicted both PANSS trajectory and functional ratings at three-year follow-up extends this mechanistic narrative into the prognostic domain, suggesting that serial MMN assessment could alert clinicians to neurophysiological deterioration preceding frank symptom relapse. The translational bridge [25]—linking MMN deficits to elevated cortisol, pro-inflammatory cytokines, and reduced gray matter volume specifically in future converters—additionally connects the neurophysiological domain to the blood-based biomarker literature, suggesting that combined monitoring of MMN and inflammatory markers might capture complementary and partially independent aspects of relapse vulnerability [28].

The P300 literature presents a more complex picture. Its state-sensitive properties—reflecting symptom fluctuations rather than stable trait characteristics in at least some paradigms, as suggested by the longitudinal correlations between P300 amplitude change and positive symptom change [28]—make it potentially more sensitive to dynamic clinical deterioration but also more susceptible to non-disease influences including antipsychotic effects, acute stress, and task engagement variability. The inter-trial variability decomposition [29] adds methodological nuance: elevated P300 variability, specifically associated with negative symptom severity and cognitive impairment in schizophrenia, may capture aspects of neural signal instability that conventional amplitude measures miss. Nevertheless, the specific predictive utility of P300 for relapse events in stabilized populations with established illness remains insufficiently characterized by the studies reviewed here, and its antipsychotic sensitivity limits its interpretive clarity in treated samples.

### 4.3. Inflammatory Biomarkers: Clinical Tractability with Interpretive Complexity

Blood-based inflammatory markers—particularly IL-6, CRP, and the cortisol awakening response—represent clinically tractable biomarker candidates because they require only standard venepuncture and can be integrated into the routine metabolic monitoring already performed in antipsychotic-treated patients. Among the studies included in this review, the most direct evidence for the prognostic relevance of these markers comes from Mondelli et al. (2015) [36], who demonstrated that non-responders to antipsychotic treatment at twelve weeks were distinguishable at the baseline by lower cortisol awakening response, higher IL-6, and higher IFN-γ, with effect sizes in the medium-to-large range and differences persisting at follow-up. The population-level developmental evidence [34], showing that elevated IL-6 in childhood prospectively predicted psychotic disorder in young adulthood in a dose-dependent manner, and the clinical evidence [35] showing a non-significant trend toward higher IL-6 in ARMS individuals who subsequently converted, together suggest that inflammatory dysregulation is not merely a state marker of acute illness but reflects a neurobiological vulnerability that precedes clinical deterioration.

Antipsychotic confounding is particularly acute for IL-6 and CRP, as discussed in Section 4.1, and was inadequately addressed in the majority of inflammatory marker studies included here. The relationship between peripheral inflammatory markers and central neuroinflammation remains incompletely established: peripheral cytokines reflect systemic immune activation that may not directly mirror neuroinflammatory processes in the brain parenchyma, and the clinical significance of modest between-group differences in circulating IL-6 for individual-level prediction remains uncertain [36,44,45]. Physical health comorbidities—metabolic syndrome, obesity, smoking, and intercurrent infections—that are highly prevalent in schizophrenia populations independently elevate inflammatory markers, introducing confounders that are frequently unaddressed in regression models [46,47,48].

Most critically, the directionality of the inflammatory–relapse relationship remains to be established, and adequately powered longitudinal studies with serial biomarker sampling are needed to resolve this question. Adequately powered longitudinal studies with serial biomarker sampling—enabling the examination of inflammatory trajectories in the weeks immediately preceding clinically identified relapse—are needed to resolve this directionality question and to establish whether inflammatory monitoring constitutes a genuine prospective warning signal or a concurrent correlate of deterioration.

It should be noted that several additional blood-based markers with established relevance to schizophrenia pathophysiology—including BDNF, NGF, IL-18, and IL-23—were not included in the present synthesis because the available studies did not meet our inclusion criteria, primarily due to cross-sectional designs, absence of prospective relapse outcomes, or non-stabilized patient populations. Their prospective evaluation in clinically stabilized patients with pre-specified relapse outcomes represents an important priority for future biomarker research in this field.

### 4.4. Digital Phenotyping: Ecological Promise, Methodological Infancy

Digital phenotyping approaches represent the most methodologically novel domain reviewed here, offering ecological validity advantages that no laboratory-based measure can replicate. The ability to continuously monitor behavioral parameters in a patient’s natural environment—capturing sleep–wake cycles, activity levels, social communication patterns, and mobility—without requiring patient initiative or clinic attendance generates a qualitatively distinct class of relapse signal. Among the studies included in this review, Adler [41] provided direct evidence that passive smartphone sensing data can detect behavioral anomalies in the thirty-day pre-relapse window, with a 108% increase in anomaly frequency relative to stable periods, and that individual-level behavioral features discriminated near-relapse from stable phases with medium-to-large effect sizes in multiply-relapsing participants. Garyfalli [42] extended this evidence to wearable sensing, demonstrating systematic associations between smartwatch-derived heart rate variability and motor activity indices and PANSS psychopathology dimension scores across more than 740 monthly assessment points, with distinct physiological signatures corresponding to positive, negative, cognitive, and affective symptom dimensions.

Yet the field remains in methodological infancy. Both reviewed studies are small—60 and 38 participants, respectively—and rely on single-site designs with heterogeneous outcome definitions, precluding direct comparison or pooled analysis. The personalization challenge is fundamental: behavioral baselines vary enormously between individuals, and clinically meaningful relapse signals must be defined relative to each person’s established patterns rather than population norms, requiring extended baseline monitoring periods before predictive models can be calibrated. This constrains applicability in early illness and newly transferred patients. Privacy concerns, digital divide disparities in smartphone and wearable ownership across age and socioeconomic groups, and technical dropout—a non-trivial obstacle over the multi-month follow-up periods required—further limit generalizability. Whether digital sensing adds predictive value incrementally beyond structured symptom self-monitoring administered through the same smartphone platform remains to be established in adequately powered head-to-head comparison studies.

### 4.5. Limitations and Future Priorities

Several important methodological limitations characterize the existing relapse biomarker literature synthesized in this review. First, as detailed in Section 4.1, antipsychotic confounding is inadequately addressed in the majority of included studies, leaving open the possibility that reported biomarker associations primarily reflect pharmacological rather than neurobiological vulnerability signals. Future studies should adopt designs that explicitly model medication type, dose, adherence, and treatment duration as covariates, or examine biomarker validity in naturalistic antipsychotic dose-reduction or discontinuation contexts with appropriate prospective controls. Second, a substantial proportion of included studies were conducted in CHR or first-episode populations rather than in fully stabilized patients with established illness—the population most directly relevant to maintenance treatment decisions—reflecting the genuine scarcity of prospective longitudinal biomarker data in the latter group. Dedicated biomarker studies in stabilized established-illness populations, with adequate follow-up periods and pre-specified relapse criteria, represent the most critical gap in the current literature. Third, relapse definitions are highly heterogeneous across studies—ranging from hospitalization records to structured psychopathology rating scale thresholds to clinician consensus judgment—making cross-study comparison problematic and limiting the development of clinically actionable prediction rules. Adoption of standardized relapse criteria consistent with validated operational definitions should become the field standard. Fourth, the near-exclusive focus on single-biomarker domains in individual studies misses the likely multimodal structure of relapse vulnerability, in which neurophysiological, inflammatory, neuroendocrine, and behavioral signals may capture complementary aspects of a common underlying destabilization process.

Future research priorities should address these limitations through specific methodological advances. Adequately powered multi-site studies with standardized protocols, prospective registration of analysis plans, and explicit antipsychotic confounding controls are urgently needed. Serial biomarker sampling at multiple timepoints—enabling trajectory analysis and change-score modeling rather than single-baseline assessment—should replace the dominant single-timepoint design that characterized the majority of studies reviewed here. Multimodal studies examining combinations of ERP measures, inflammatory markers, and digital behavioral signals within the same participants and follow-up period would enable the incremental validity analysis necessary to determine whether different biomarker domains capture independent versus redundant relapse-related variance. Finally, tri-level outcome classifications distinguishing frank relapse, persistent symptomatic burden without acute decompensation, and genuine clinical remission would prevent the conflation of heterogeneous non-relapsing outcomes that artificially inflates biomarker effect sizes in dichotomized analyses.

### 4.6. Limitations of the Review Process

Several limitations of the review process itself warrant acknowledgment. First, although five databases were searched with no language restrictions, publication bias cannot be excluded: studies reporting null or negative biomarker findings are less likely to be published, potentially inflating the apparent strength of associations reported here. Second, the substantial methodological heterogeneity across included studies—in populations, biomarker domains, outcome definitions, and follow-up durations—precluded quantitative meta-analysis, limiting our ability to derive pooled effect estimates or formally test sources of heterogeneity through meta-regression. The narrative synthesis adopted here, albeit appropriate for this heterogeneity, is inherently more susceptible to interpretive subjectivity than quantitative pooling. Third, the review was conducted by a relatively small team, and despite the use of independent dual screening, the risk of selective interpretation cannot be entirely eliminated. Finally, the rapid evolution of the digital phenotyping and molecular biomarker fields means that relevant studies published after March 2026 are not captured in this synthesis.

The present findings should be interpreted alongside a recently published systematic review by Smyrnis et al. [20], which similarly synthesized biomarker evidence for psychotic relapse prediction across genetic, blood-based, neuroimaging, cognitive–behavioral, and wearable domains. Areas of convergence between the two reviews—including the state-marker candidacy of IL-6, the failure of neuroimaging models to achieve prospective predictive accuracy, and the ecological promise but methodological immaturity of digital phenotyping—may be interpreted as independent corroboration of these conclusions across reviews with partially distinct methodological approaches. The most substantive divergence concerns neurophysiological biomarkers, a domain entirely absent from Smyrnis et al., yet yielding the most consistent evidence in the present synthesis, with MMN demonstrating a degree of independence from antipsychotic effects that distinguishes it from virtually all other candidate markers. The convergent findings strengthen confidence in shared conclusions, while the inclusion of neurophysiological biomarkers and the IL-6/cortisol awakening response combination as a multimodal candidate represent the most distinct contributions of the present review.

### 4.7. Toward Clinical Implementation: What Is Needed?

Translating relapse biomarkers into clinical practice requires substantially more than establishing statistical associations in research cohorts. For a biomarker to earn a place in clinical monitoring protocols, it must satisfy four conditions: (1) sufficient predictive accuracy, with positive predictive values, sensitivity, and specificity adequate to guide clinically meaningful decisions at the individual level rather than group averages; (2) incremental predictive value beyond routine clinical assessment, since structured symptom monitoring, adherence measurement, and clinician judgment already provide substantial prognostic information whose incremental improvement by any biomarker must be explicitly demonstrated; (3) feasibility and acceptability in real-world psychiatric settings, encompassing measurement cost, technical infrastructure requirements, training burden, and patient willingness to undergo repeated assessment; and (4) demonstrated impact on clinical decision-making, and ultimately, on patient outcomes through prospective biomarker-guided intervention trials rather than retrospective observational inference.

By these standards, no biomarker reviewed here currently qualifies for implementation in routine clinical practice. The most mature candidates—MMN for neurophysiological monitoring and the IL-6/CRP/cortisol awakening response combination for blood-based monitoring—represent reasonable targets for proof-of-concept clinical implementation trials in specialized early psychosis services or antipsychotic dose-reduction research contexts, where the clinical question is sufficiently well-defined and the monitoring infrastructure sufficiently developed. Digital phenotyping, despite its ecological validity advantages, requires the resolution of fundamental privacy and data governance concerns, equity of access across the socioeconomic diversity of psychosis populations, and rigorous external validation across diverse clinical settings before widespread implementation can be responsibly recommended. The field’s most honest current contribution to clinical practice may be its principal negative finding: no biomarker can currently reliably identify which stabilized patients can safely reduce or discontinue antipsychotic treatment—a question of profound clinical and ethical importance that demands dedicated biomarker-guided discontinuation trials, not extrapolation from the heterogeneous observational literature reviewed here.

## 5. Conclusions

This systematic review documents a field at an important transitional moment: sufficiently mature to identify converging evidence for several candidate biomarker domains, yet insufficiently advanced to deliver clinically implementable prediction tools for psychotic relapse. The evidence base for neurophysiological markers—particularly MMN as a trait-sensitive measure of glutamatergic circuit integrity and P300 as a state-sensitive index of attentional processing—represents the most methodologically rigorous and mechanistically grounded domain, with serial trajectory assessment showing particular promise over single-baseline measurement. Inflammatory and neuroendocrine biomarkers offer practical clinical tractability but require the resolution of antipsychotic confounding challenges and directionality questions before their predictive utility can be confidently established. Gene expression profiling has opened a genuinely novel molecular window into relapse pathobiology, with the TCF4 and ubiquitin-proteasome findings from the 2EPs project providing a compelling research direction. Digital phenotyping approaches, while methodologically nascent, capture an ecological dimension of relapse vulnerability invisible to laboratory-based biomarkers and may ultimately provide the continuous monitoring infrastructure into which other biomarker signals can be integrated. The honest assessment required of this field is that the relapse prediction challenge is harder than the CHR-to-psychosis transition question—not less important, but harder. Every biological signal is confounded by treatments that alter the very systems under study. The outcome is multiply determined by adherence, stress exposure, comorbid substance use, and social determinants that laboratory measures cannot capture. The patients are heterogeneous across illness duration, treatment history, and illness subtype in ways that population-average biomarker findings obscure. Additionally, the clinical stakes—potentially exposing patients to relapse risk or to indefinite antipsychotic treatment burden—are substantial. These challenges demand rigorous methodological responses, not dampened scientific ambition. A young person stabilized on antipsychotics after a psychotic episode, wondering whether they can safely reduce or stop their medication, deserves our best scientific efforts to answer that question with evidence rather than convention. Biomarkers that could genuinely individualize maintenance treatment decisions—telling us whose dopaminergic vulnerability has normalized versus those whose neuroinflammatory dysregulation persists, whose behavioral ecology shows stable resilience versus emerging destabilization—could transform the long-term management of psychotic disorders. The path to those biomarkers runs through the methodological advances identified here: multimodal longitudinal designs, antipsychotic-confounding controls, standardized relapse definitions, and the humility to report null findings alongside positive ones. The field’s current mandate is to build the rigorous evidence base that patients and clinicians deserve.

Based on the evidence synthesized, we propose a theoretical Biomarker-Informed Monitoring Protocol as a framework for future trial design. In patients stabilized on maintenance antipsychotic treatment—particularly those considered for dose reduction—we suggest: (1) baseline assessment of MMN amplitude and serum IL-6 and cortisol awakening response; (2) reassessment at 3-month intervals to capture biological trajectories rather than single timepoints; (3) continuous passive digital monitoring via smartphone or wearable device to detect behavioral anomalies in the intervals between clinic visits; and (4) a pre-specified biological alert threshold—defined as a clinically meaningful deviation from the individual baseline in two or more of these domains—triggering clinical reassessment, and if indicated, treatment intensification. This protocol is not proposed for immediate implementation but as a structured hypothesis for prospective biomarker-guided intervention trials.

## Figures and Tables

**Table 1:** Methodological quality assessment of included studies (Newcastle-Ottawa Scale and PROBAST).

First Author	Biomarker Domain	Tool Used	Score/Rating	APD (Antipsychotic Drug) Confounding Controlled?	Relapse Definition Standardized?	Adherence Measured/ Controlled?	Validation	Key Quality Issues
Bodatsch et al. [19]	Neurophysiological (MMN)	NOS	7 (High)	Yes (antipsychotic-naive)	Yes (BLIPS/CAARMS criteria)	Not applicable (naive)	None (single site, prospective)	Small N (n = 62); no external validation
Hamilton et al. [23]	Neurophysiological (MMN)	NOS + PROBAST	8 (High)/Low risk	Partial (unmedicated subsample)	Yes (SIPS/CAARMS)	Partial	Cross-site leave-one-out (NAPLS-2)	Medication modulates MMN; subsample analysis limits generalizability
Nakajima et al. [24]	Neurophysiological (dMMN/fMMN)	NOS	5 (Moderate)	No	Partial (PANSS threshold)	Not reported	None	Very small N (n = 30); no independent validation; remission not relapse
Hamilton et al. [25]	Neurophysiological + Inflammatory	NOS + PROBAST	7 (High)/Low risk	Partial	Yes (SIPS/CAARMS)	Partial	Cross-site (NAPLS-2 subsample)	Subsample for bloods (n = 57); CHR population only
Giordano et al. [26]	Neurophysiological (MMN, P3a)	NOS	5 (Moderate)	Not addressed	Not applicable (functioning)	Not applicable	None	Cross-sectional; no relapse outcome; multicenter but cross-sectional design
Light & Braff [27]	Neurophysiological (MMN)	NOS	4 (Moderate)	Not addressed	Not applicable (functioning proxy)	Not reported	None	N = 10 per group; pilot; functioning outcome only
Higashima et al. [28]	Neurophysiological (P300)	NOS	5 (Moderate)	Not addressed (treated)	Partial (PANSS change)	Not reported	None	Mixed cross-sectional and longitudinal; P300 state-sensitive; medication not controlled
Kim et al. [29]	Neurophysiological (P300-ITV)	NOS	4 (Moderate)	Not addressed	Not applicable (cross-sectional)	Not applicable	None	Cross-sectional; novel decomposition without longitudinal validation
De Wilde et al. [30]	Neurophysiological (P300)	NOS	5 (Moderate)	Not addressed	Not applicable (cross-sectional)	Not applicable	None	Cross-sectional endophenotype study; no relapse prediction
Van Tricht et al. [31]	Neurophysiological (P50, N1, P2)	NOS + PROBAST	6 (Moderate)/Moderate risk	Mixed (some medication-naive)	Yes (CAARMS)	Not reported	None	Small converter subgroup (n = 18); gating measures less stable than MMN
Duncan et al. [32]	Neurophysiological (N100)	NOS + PROBAST	8 (High)/Low risk	Partial	Yes (SIPS/CAARMS)	Partial	Cross-site (NAPLS-2)	CHR population; extends MMN literature to N100
Brockhaus-Dumke et al. [33]	Neurophysiological (P50, N100)	NOS	7 (High)	Yes (antipsychotic-free or naive)	Yes (BLIPS/CAARMS)	Not applicable (naive/free)	None	Negative finding for gating as conversion predictor; important null result
Khandaker et al. [34]	Inflammatory (IL-6, CRP)	NOS	8 (High)	Yes (population cohort, drug-naive)	Yes (ICD-10 at age 18)	Not applicable (population)	External (birth cohort, population representative)	Population cohort; childhood inflammatory exposure; psychosis not stabilized relapse
Stojanovic et al. [35]	Inflammatory (IL-6, CRP)	NOS	5 (Moderate)	Not addressed	Partial (clinical assessment)	Not reported	None	Very small ARMS group (n = 17); underpowered for conversion comparison
Mondelli et al. [36]	Inflammatory + Neuroendocrine	NOS + PROBAST	7 (High)/Low risk	Partial (naive at baseline)	Partial (structured at 12 weeks)	Partial	None (single site)	Short follow-up (12 weeks); treatment response not relapse per se
Gassó et al. [37]	Molecular (gene expression)	NOS + PROBAST	7 (High)/Moderate risk	Partial (treatment documented)	Partial (clinical)	Partial	Internal (bootstrap); no external cohort	Novel WGCNA approach; no external validation; 2EPs single center
Pawelczyk et al. [38]	Molecular (telomere length)	NOS	4 (Moderate)	Not addressed	Not applicable (cross-sectional)	Not applicable	None	Cross-sectional at acute exacerbation; chronicity marker, not prospective predictor
Landi et al. [39]	Molecular (PRS)	NOS + PROBAST	8 (High)/Low risk	Not applicable (genomic)	Partial (clinical outcomes)	Not applicable	External (two multi-ethnic cohorts)	Negative finding; large N; demonstrates PRS does not add over clinical variables
De Nijs et al. [40]	Neuroimaging (ML, multimodal)	NOS + PROBAST	7 (High)/Low risk	Partial (APD use as predictor)	Yes (structured GAF)	Partial	Leave-one-site-out cross-validation	Machine learning; no neuroimaging predictor survived; highlights clinical variable dominance
Adler et al. [41]	Digital phenotyping	NOS	6 (Moderate)	Not applicable (behavioral)	Partial (clinical consensus)	Not applicable	None (single study)	Small relapsing group (n = 18); high IQR (interquartile range;) personalization challenge
Garyfalli et al. [42]	Digital phenotyping (smartwatch)	NOS	6 (Moderate)	Not applicable (physiological sensing)	Partial (PANSS monthly)	Not applicable	None	Small N (n = 38); dimensional not event outcomes; wearable compliance issues

For a complete list of abbreviations, see the List of Abbreviations section.

**Table 2:** Characteristics of the included studies.

First Author (Year)	Population	Population Stage	N	Diagnosis	Biomarker Domain	Follow-Up	Relapse Definition	Primary Outcome	Key Finding
Bodatsch et al. [19]	CHR (antipsychotic-naive)	CHR	62	At-risk mental state	Neurophysiological (MMN)	32 mo (median)	Transition to psychosis	Psychosis conversion	Reduced duration MMN in converters vs. non-converters; Cox model stratified two risk classes with different survival curves
Hamilton et al. [32]	CHR-P + HC (NAPLS-2, multisite)	CHR	580 CHR + 241 HC	CHR for psychosis	Neurophysiological (MMN)	24 mo	Transition to psychosis (SIPS/CAARMS)	Psychosis conversion (n = 77)	MMN reduced in converters (d = 0.27–0.43); double-deviant MMN predicted earlier conversion (HR = 1.40; 95% CI 1.03–1.90) in unmedicated subsample
Nakajima et al. [24]	First-episode schizophrenia + HC	FEP	30 + 22 HC	Schizophrenia (first episode)	Neurophysiological (dMMN/fMMN)	~3 years	Symptomatic remission (PANSS threshold)	Symptomatic remission	Non-remitters showed lower baseline dMMN amplitude and prolonged latency; baseline dMMN predicted PANSS and SCoRS scores at follow-up
Hamilton et al. [25]	CHR-P (NAPLS-2 subsample)	CHR	303 (57 with blood draws)	CHR for psychosis	Neurophysiological + Inflammatory (MMN, cortisol, cytokines)	24 mo	Transition to psychosis (SIPS/CAARMS)	Psychosis conversion	Deficient MMN correlated with higher cortisol, pro-inflammatory cytokines, and smaller gray matter volume specifically in future converters
Giordano et al. [26]	Established schizophrenia (4 illness duration groups) + HC	Established SZ	117 + 61 HC	Schizophrenia (ICD/DSM)	Neurophysiological (p-MMN, d-MMN, P3a)	Cross-sectional (functioning outcomes)	Real-life functioning (SFS)	Functional outcomes	MMN reduced regardless of illness duration; p-MMN linked to Work skills domain; P3a reduced only in the longest-duration group (19–32 years)
Light & Braff [27]	Chronic schizophrenia + HC	Established SZ	10 + 10 HC	Chronic schizophrenia	Neurophysiological (MMN)	1–2 years (2 assessments)	Functional status (longitudinal proxy)	Functional status	MMN deficits stable across both timepoints with large effect sizes; associated with poor functioning at both assessments; symptom ratings less consistent
Higashima et al. [28]	Schizophrenia/schizophreniform	Established SZ	93 (cross-sect.) + 20 (longit.)	Schizophrenia/schizophreniform	Neurophysiological (P300)	~238 days (longitudinal)	Positive symptom change (PANSS)	Change in positive syndrome scores	P300 correlated negatively with positive symptoms cross-sectionally; ΔP300 correlated with Δpositive symptoms longitudinally; left posterior temporal strongest
Kim et al. [29]	Schizophrenia, GHR, CHR, HC	Mixed	45 SZ + 32 GHR + 32 CHR + 52 HC	Schizophrenia; high-risk groups	Neurophysiological (P300 inter-trial variability)	Cross-sectional	Not applicable (cross-sectional)	Group differences in P300 components; negative symptoms; cognition	ITV elevated specifically in CHR and SZ, not in GHR or HC; higher ITV associated with more negative symptoms and worse cognition in the SZ group
De Wilde et al. [30]	First-episode schizophrenia + siblings + HC	FEP	53 FEP + 27 siblings + 28 HC	First-episode schizophrenia	Neurophysiological (P300)	Cross-sectional	Not applicable (cross-sectional)	Endophenotype assessment	P300 amplitude reduced in patients but not in unaffected siblings relative to controls; P300 latency did not differ across groups
Van Tricht et al. (2015) [31]	Ultra-high risk (18 converters) + HC	CHR	61 UHR + 28 HC	UHR for psychosis	Neurophysiological (P50, N1, P2 gating)	18 mo (2 assessments)	Transition to psychosis (CAARMS)	Psychosis conversion	Smaller N1 difference score in converters at baseline; post-conversion reductions in N1 and P2; gating modestly predictive of transition
Duncan et al. [32]	CHR (NAPLS-2)	CHR	552 CHR + 236 HC	CHR for psychosis	Neurophysiological (N100)	24 mo	Transition to psychosis (SIPS/CAARMS)	Psychosis conversion (n = 73)	Smaller N100 at Cz in converters; predicted conversion likelihood and shorter time-to-conversion for standard and novel stimuli independently
Brockhaus-Dumke et al. [33]	At-risk, prodromal, FEP, chronic SZ, HC	Mixed	18 AR + 21 prodromal + 46 FEP + 20 chronic + 46 HC	CHR; first-episode; chronic schizophrenia	Neurophysiological (P50, N100 gating)	~2 years (converters)	Transition to psychosis (BLIPS/CAARMS)	Psychosis conversion (truly prodromal group)	P50 impaired across all clinical groups; N100 suppression reduced only in prodromal and FEP; at-risk converters vs. non-converters: no significant difference on any gating parameter
Khandaker et al. [34]	Population birth cohort (ALSPAC)	General population	~4500	General population (psychosis at age 18)	Blood-based inflammatory (IL-6, CRP)	~9 years (age 9 to 18)	Psychotic disorder or experiences at age 18 (ICD-10)	Psychotic outcomes at age 18	Top tertile IL-6 at age 9: OR 1.81 for psychotic experiences (95% CI 1.01–3.28); OR 2.40 for psychotic disorder (0.88–6.22); CRP not independently predictive after full adjustment
Stojanovic et al. [35]	ARMS + psychotic disorder + HC	Mixed	17 ARMS + 77 psychosis + 25 HC	At-risk; psychotic disorder (ICD-10)	Blood-based inflammatory (IL-6, CRP, fibrinogen)	26 mo	Transition to psychosis (in ARMS group)	Psychosis conversion (6/17 ARMS)	Higher IL-6 in ARMS vs. HC (persistent after excluding cannabis users); converters showed higher median IL-6 (0.61 vs. 0.35 pg/mL)—non-significant (underpowered); IL-6 correlated with negative symptoms
Mondelli et al. [36]	First-episode psychosis + HC	FEP	68 FEP + 57 HC	First-episode psychosis (DSM-IV)	Blood-based inflammatory + neuroendocrine (cortisol, IL-6, IFN-γ)	12 weeks	Treatment response (structured assessment at 12 weeks)	Response vs. non-response at 12 weeks	Non-responders: lower cortisol awakening response (d = 0.6), higher IL-6 (d = 1.0), higher IFN-γ (d = 0.9) at baseline; differences persisted at 12-week follow-up
Gassó et al. [37]	First-episode schizophrenia (2EPs Project)	FEP	91 baseline; 67 follow-up	Schizophrenia (first-episode)	Molecular (gene expression—WGCNA)	3 years stable or at relapse	Relapse (structured clinical assessment)	Relapse vs. 3-year stability	DarkTurquoise module (TCF4 network) dysregulated at relapse; DarkRed baseline expression associated with greater relapse risk and earlier onset (*p* = 0.045); ubiquitin-proteasome pathway implicated
Pawelczyk et al. [38]	Early + chronic schizophrenia	Established SZ	42 early + 44 chronic	Schizophrenia (ICD-10)	Molecular (telomere length)	Cross-sectional (acute exacerbation)	Not applicable (cross-sectional; correlates of chronicity)	Symptom severity; episode count; hospitalizations	Telomere length correlated with symptom severity, number of episodes, and hospitalizations; regression model (illness group, sex, age, episode burden) explained R^2^ = 0.512 of variance
Landi et al. [39]	Two multi-ethnic cohorts	Established SZ	8541	Adults with psychotic disorder	Molecular (polygenic risk score)	Prospective (variable)	Various clinical outcomes	PRS added predictive value over clinical variables?	SZ PRS did not improve predictive model performance across any outcome or ancestral background; clinical interview variables were dominant predictors
De Nijs et al. [40]	Established psychotic illness (multicenter)	Established SZ	523	Psychotic disorder (variable duration)	Neuroimaging (machine learning on multimodal baseline data)	3 and 6 years	Symptomatic and global outcomes (GAF)	3- and 6-year symptomatic/global outcomes	Prediction accuracy 62–68%; leave-one-site-out cross-validation; only clinical variables (GAF, symptoms, antipsychotic use, QoL) emerged as dominant predictors—no neuroimaging biomarker contributed
Adler et al. [41]	Schizophrenia spectrum (CrossCheck study)	Established SZ	60 (42 non-relapsing, 18 relapsing)	Schizophrenia spectrum	Digital phenotyping (passive smartphone sensing)	Variable (20,137 person-days)	Relapse (clinical consensus assessment)	Relapse detection (30-day pre-relapse window)	Autoencoder sensitivity 0.25 (IQR 0.15–1.00), specificity 0.88 (IQR 0.14–0.96); 108% increase in behavioral anomalies in near-relapse period; individual-level features with medium-to-large effect sizes in multiply-relapsing participants
Garyfalli et al. [42]	Psychotic spectrum (e-Prevention study)	Established SZ	38	Psychotic spectrum disorders	Digital phenotyping (smartwatch passive sensing)	Up to 26 months (>740 monthly observations)	PANSS 5-factor dimension scores (monthly)	Psychopathology dimension scores	↑ Positive symptoms: ↓ HRV heart rate variability during sleep. ↑ Negative symptoms: ↓ motor activity (wakefulness). ↑ Depression/excitement: ↑ motor activity during sleep, ↑ normalized HR. ↑ Cognitive symptoms: ↓ Heart rate variability (HRV) wakefulness

For a complete list of abbreviations, see the List of Abbreviations section.

**Table 3:** Neurophysiological biomarkers: Study-level summary.

First Author	ERP Component	Population	N	Follow-Up	Outcome	Key Quantitative Finding	APD Confound Addressed?	Comment/Limitations
Bodatsch et al. [19]	Duration MMN	CHR (antipsychotic-naive)	62	32 mo	Psychosis conversion	Converters < non-converters at frontocentral electrodes; Cox model: two risk classes with different survival curves	Yes (drug-naive)	Landmark CHR study; small N; no stabilized SZ population
Hamilton et al. [25]	MMN (duration, frequency, double-deviant)	CHR-P (NAPLS-2, multisite)	821	24 mo	Psychosis conversion (n = 77)	d = 0.27 (full sample); d = 0.43 (unmedicated, double-deviant); HR = 1.40 (95% CI 1.03–1.90) in unmedicated subsample	Partial (unmedicated subsample analyzed separately)	Largest prospective MMN study; multisite; medication modulates effect size
Nakajima et al. [24]	dMMN, fMMN	First-episode schizophrenia	30 + 22 HC	~3 years	Symptomatic remission	Non-remitters lower dMMN amplitude and prolonged latency at baseline; baseline dMMN predicted PANSS and SCoRS (logistic regression)	Not explicitly	Small N; no independent validation; remission not relapse as outcome
Hamilton et al. [25]	MMN (multimodal)	CHR-P (NAPLS-2 subsample)	303 (57 with bloods)	24 mo	Psychosis conversion	Deficient MMN correlated with higher cortisol, IL-6, smaller gray matter volume in future converters only	Partial	Integrative cross-domain study; links MMN to inflammation and structure; CHR not stabilized SZ
Giordano et al. [26]	p-MMN, d-MMN, P3a	Established schizophrenia (4 illness duration groups)	117 + 61 HC	Cross-sectional (functioning)	Real-life functioning (SFS)	MMN reduced regardless of duration; p-MMN specifically associated with ‘Work skills’ domain; P3a reduced only in longest duration group	Not addressed	Cross-sectional; no relapse outcome; functioning proxy
Light & Braff [27]	MMN	Chronic schizophrenia	10 + 10 HC	1–2 years (2 timepoints)	Functional status	Large effect sizes stable across both timepoints; MMN predicted functioning at both assessments; symptom ratings less consistent	Not addressed (chronic, treated)	Very small N; no formal relapse prediction; demonstrates trait-stability of MMN
Higashima et al. [28]	Auditory P300	Schizophrenia/schizophreniform	93 (X-sect.) + 20 (longit.)	~238 days (longitudinal)	Change in positive syndrome scores	P300 correlated negatively with positive symptoms cross-sectionally; ΔP300 correlated with ΔPositive symptoms longitudinally; left posterior temporal strongest	Not addressed (treated)	P300 state-sensitive; medication effects likely; positive symptoms only
Kim et al. [29]	P300 (amplitude + inter-trial variability)	SZ, GHR, CHR, HC	161 total	Cross-sectional	Group comparison; negative symptoms; cognition	ITV elevated in CHR and SZ, not in GHR or HC; higher ITV associated with more negative symptoms and worse cognition in SZ group	Not addressed	Cross-sectional; ITV as novel P300 decomposition; no relapse or follow-up data
De Wilde et al. [30]	P300 (amplitude + latency)	FEP + siblings + HC	108 total	Cross-sectional	Endophenotype (group comparison)	P300 reduced in patients, not in unaffected siblings; latency did not differ	Not addressed	Cross-sectional; endophenotype focus; no longitudinal or relapse outcome
Van Tricht et al. [31]	P50, N1, P2 gating	UHR (18 converters)	61 UHR + 28 HC	18 mo (2 assessments)	Psychosis conversion	Smaller N1 difference score in converters at baseline; post-conversion reductions in N1 and P2; gating modestly predictive	Not addressed (mixed medication status)	Small N in converter subgroup; gating less robust than MMN as predictor
Duncan et al. [32]	N100	CHR-P (NAPLS-2)	788 total	24 mo	Psychosis conversion (n = 73)	Smaller N100 at Cz predicted conversion likelihood and shorter time-to-conversion for standard and novel stimuli independently	Partial	Large multisite study; N100 as complement to MMN; converters identified prospectively
Brockhaus-Dumke et al. [33]	P50, N100 gating	AR, truly prodromal, FEP, chronic SZ, HC	151 total	~2 years (converters)	Psychosis conversion	P50 impaired across all clinical groups; N100 suppression reduced in prodromal and FEP; at-risk converters vs. non-converters: no significant difference on any parameter	Yes (antipsychotic-free or naive groups)	Gating did not discriminate CHR converters; highlights limits of gating for transition prediction

For a complete list of abbreviations, see the List of Abbreviations section.

**Table 4:** Blood-based, molecular, neuroimaging, and digital phenotyping biomarkers: study-level summary.

First Author	Biomarker Domain	Population	N	Follow-Up	Outcome	Key Quantitative Finding	APD Confound Addressed?	Comment/Limitations
Khandaker et al. [34]	Inflammatory (IL-6, CRP)	Population birth cohort (ALSPAC)	~4500	~9 years	Psychotic experiences/disorder at 18	Top tertile IL-6 at age 9: OR 1.81 for psychotic experiences (95% CI 1.01–3.28); OR 2.40 for psychotic disorder (0.88–6.22); CRP not independently predictive	Yes (drug-naive; population sample)	Population cohort; drug-naive; childhood IL-6 measured; psychosis not remission/relapse context
Stojanovic et al. [35]	Inflammatory (IL-6, CRP, fibrinogen)	ARMS + psychotic disorder + HC	17 ARMS + 77 psychosis + 25 HC	26 mo	Transition (in ARMS)	Higher IL-6 in ARMS vs. HC (persistent after excluding cannabis users); converters (6/17) showed higher median IL-6 (0.61 vs 0.35 pg/mL)—non-significant (underpowered)	Not addressed	Very small ARMS subgroup (n = 17); non-significant conversion comparison; IL-6 correlated with negative symptoms
Mondelli et al. [36]	Inflammatory + neuroendocrine (cortisol, IL-6, IFN-γ)	First-episode psychosis	68 FEP + 57 HC	12 weeks	Treatment response (responders vs. non-responders)	Non-responders: lower CAR (cortisol awakening response) (d = 0.6, *p* = 0.03); higher IL-6 (d = 1.0, *p* = 0.003); higher IFN-γ (d = 0.9, *p* = 0.02); differences persisted at 12 weeks	Partial (antipsychotic-naive at baseline)	Treatment response not relapse per se; 12-week follow-up relatively short; inflammatory and HPA axis markers complementary
Gassó et al. [37]	Molecular (gene expression—WGCNA)	First-episode schizophrenia (2EPs)	91 baseline; 67 follow-up	3 years/at relapse	Relapse vs. 3-year stable	DarkTurquoise module (TCF4 network) semi-conserved at relapse; DarkRed baseline expression associated with relapse risk and earlier onset (*p* = 0.045); ubiquitin-proteasome pathway implicated	Partial (antipsychotic treatment documented)	Novel molecular approach; co-expression network analysis; no external validation; 2EPs single-center cohort
Pawelczyk et al. [38]	Molecular (telomere length)	Early + chronic schizophrenia	42 early + 44 chronic	Cross-sectional (acute)	Chronicity markers (episode count, hospitalizations)	Telomere length correlated with severity, episodes, hospitalizations; regression model R^2^ = 0.512 incorporating illness group, sex, age, episode burden	Not addressed (treated)	Cross-sectional; acute exacerbation context; telomere length as chronicity not prospective relapse predictor
Landi et al. [39]	Molecular (polygenic risk score)	Two multi-ethnic cohorts	8541	Prospective (variable)	Various clinical outcomes	SZ PRS did not improve predictive model performance over clinical interview variables in any outcome or ancestral background	Not applicable (genomic)	Negative finding; largest genomic study reviewed; PRS may capture lifetime risk not state-dependent relapse vulnerability
De Nijs et al. [40]	Neuroimaging (ML on multimodal data)	Established psychotic illness (multicenter)	523	3 and 6 years	Symptomatic + global outcomes (GAF)	Accuracy 62.2–64.7% (symptomatic); 63.5–67.6% (global); leave-one-site-out CV; only clinical variables (GAF, symptoms, antipsychotic use, QoL) emerged—no neuroimaging predictor contributed	Not addressed	Machine learning approach; no neurobiological variable survived feature elimination; highlights limits of neuroimaging for individualized prediction
Adler et al. [41]	Digital phenotyping (passive smartphone sensing)	Schizophrenia spectrum (CrossCheck)	60 (42 non-relapsing, 18 relapsing)	Variable (20,137 person-days)	Relapse (clinical assessment)	Autoencoder: sensitivity 0.25 (IQR 0.15–1.00), specificity 0.88 (IQR 0.14–0.96); 108% increase in behavioral anomalies in near-relapse window; individual-level features with medium-to-large effect sizes in multiply-relapsing participants	Not applicable (behavioral monitoring)	Small relapsing group (n = 18); wide IQR indicates high individual variability; personalization approach needed
Garyfalli et al. [42]	Digital phenotyping (smartwatch passive sensing)	Psychotic spectrum (e-Prevention)	38	Up to 26 months (>740 monthly observations)	PANSS 5-factor dimension scores (monthly)	↑ Positive symptoms: ↓ HRV during sleep. ↑ Negative symptoms: ↓ motor activity (wakefulness). ↑ Depression/excitement: ↑ motor activity during sleep, ↑ normalized HR sleep. ↑ Cognitive symptoms: ↓ HRV wakefulness	Not applicable (physiological sensing)	Small N; dimensional outcomes not relapse events; no external validation; long-term wearable compliance challenging

For a complete list of abbreviations, see the List of Abbreviations section.

## Data Availability

No new data were created or analyzed in this study. Data sharing is not applicable to this article.

## Abbreviations

**ALSPAC**: Avon Longitudinal Study of Parents and Children; **APD**: antipsychotic drug; **ARMS**: At-Risk Mental State; **BLIPS**: Brief Limited Intermittent Psychotic Symptoms; **BMI**: body mass index; **CAARMS**: Comprehensive Assessment of At-Risk Mental States; **CAR**: cortisol awakening response; **CHR**: clinical high-risk for psychosis; **CI**: confidence interval; **CRP**: C-reactive protein; **dMMN**: duration mismatch negativity; **DSM**: Diagnostic and Statistical Manual of Mental Disorders; **EEG**: electroencephalography; **ERP**: event-related potential; **FEP**: first-episode psychosis; **fMMN**: frequency mismatch negativity; **GAF**: Global Assessment of Functioning; **GHR**: genetic high risk; **HC**: healthy control; **HPA**: hypothalamic–pituitary–adrenal; **HR**: hazard ratio; **HRV**: heart rate variability; **ICD**: International Classification of Diseases; **IFN-γ**: interferon-gamma; **IL-6**: interleukin-6; **IQR**: interquartile range; **ITV**: inter-trial variability; **ML**: machine learning; **MMN**: mismatch negativity; **MRI**: magnetic resonance imaging; **N100**: N100 event-related component; **NAPLS-2**: North American Prodrome Longitudinal Study-2; **NMDA**: N-methyl-D-aspartate; **NOS**: Newcastle-Ottawa Scale; **OR**: odds ratio; **P300**: P300 event-related potential; **p-MMN**: pitch mismatch negativity; **PANSS**: Positive and Negative Syndrome Scale; **PRISMA**: Preferred Reporting Items for Systematic Reviews and Meta-Analyses; **PROBAST**: Prediction Model Risk of Bias Assessment Tool; **PROSPERO**: International Prospective Register of Systematic Reviews; **PRS**: polygenic risk score; **QoL**: quality of life; **SCoRS**: Schizophrenia Cognition Rating Scale; **SFS**: Social Functioning Scale; **SIPS**: Structured Interview for Psychosis-Risk Syndromes; **SZ**: schizophrenia; **TCF4**: transcription factor 4; **TNF-α**: tumor necrosis factor-alpha; **UHR**: ultra-high risk; **WGCNA**: weighted gene co-expression network analysis.

## Appendix A

**Table A1:** PRISMA 2020 checklist.

Section/Topic	Item	Checklist Item	Reported	Location in Manuscript
***TITLE***				
**Title**	1	Identify the report as a systematic review.	**Yes**	Title page
***ABSTRACT***				
**Abstract**	2	See the PRISMA 2020 for Abstracts checklist.	**Yes**	Abstract
***INTRODUCTION***				
**Rationale**	3	Describe the rationale for the review in the context of existing knowledge.	**Yes**	Introduction, paragraphs 3–5
**Objectives**	4	Provide an explicit statement of the objective(s) or question(s) the review addresses.	**Yes**	Introduction, final paragraph
***METHODS***				
**Eligibility criteria**	5	Specify the inclusion and exclusion criteria for the review and how studies were grouped for the syntheses.	**Yes**	Section 2.1
**Information sources**	6	Specify all databases, registers, websites, organizations, reference lists and other sources searched or consulted to identify studies. Specify the date when each source was last searched or consulted.	**Yes**	Section 2.1 (PubMed, Scopus, Web of Science, PsycINFO, Embase; through March 2026)
**Search strategy**	7	Present the full search strategies for all databases and registers, including any filters applied, so that they could be repeated.	**Yes**	Section 2.1 (terms listed); full string in Table A2
**Selection process**	8	Specify the methods used to decide whether a study met the inclusion criteria of the review, including how many reviewers screened each record and each report retrieved for eligibility, whether they worked independently, and if applicable, details of automation tools used in the process.	**Yes**	Section 2.1 (two independent reviewers V.R., L.R.; disagreements resolved by G.Ma.)
**Data collection process**	9	Specify the methods used to collect data from reports, including how many reviewers collected data from each report, whether they worked independently, any processes for obtaining or confirming data from study investigators, and if applicable, details of automation tools used in the process.	**Yes**	Section 2.1 (standardized extraction forms; two reviewers)
**Data items**	10a	List and define all outcomes for which data were sought. Specify whether all results that were compatible with each outcome domain in each study were sought, and if not, the methods used to decide which results to collect.	**Yes**	Section 2.1
**Data items**	10b	List and define all other variables for which data were sought (e.g., participant and intervention characteristics, funding sources).	**Yes**	Section 2.1
**Study risk of bias assessment**	11	Specify the methods used to assess risk of bias in the included studies, including details of the tool(s) used, how many reviewers assessed each study and whether they worked independently, and if applicable, details of automation tools used in the process.	**Yes**	Section 2.2 (NOS and PROBAST; domain-specific quality criteria)
**Effect measures**	12	Specify for each outcome the effect measure(s) (e.g., risk ratio, mean difference) used in the synthesis or presentation of results.	**Yes**	Section 2.3 (narrative synthesis; effect sizes reported where available)
**Synthesis methods**	13a	Describe the processes used to decide which studies were eligible for each synthesis (e.g., tabulating the study intervention characteristics and comparing against the planned groups specified in the eligibility criteria).	**Yes**	Section 2.3
**Synthesis methods**	13b	Describe any methods required to prepare the data for presentation or synthesis, such as handling of missing summary statistics, or data conversions.	**Yes**	Section 2.3
**Synthesis methods**	13c	Describe any methods used to tabulate or visually display results of individual studies and syntheses.	**Yes**	Section 2.3; Table 2 and Table 3
**Synthesis methods**	13d	Describe any methods used to synthesize results and provide a rationale for the choice(s). If meta-analysis was performed, describe the model(s), method(s) to identify the presence and extent of statistical heterogeneity, and software package(s) used.	**Yes**	Section 2.3 (narrative synthesis chosen due to substantial methodological heterogeneity)
**Synthesis methods**	13e	Describe any methods used to explore possible causes of heterogeneity among study results (e.g., subgroup analysis, meta-regression).	**Yes**	Section 2.3 (heterogeneity discussed narratively; no meta-regression feasible)
**Synthesis methods**	13f	Describe any sensitivity analyses conducted to assess robustness of the synthesized results.	**N/A**	Not applicable (narrative synthesis)
**Reporting bias assessment**	14	Describe any methods used to assess risk of bias due to missing results in a synthesis (arising from reporting biases).	**Partial**	Section 4.5; publication bias discussed narratively
**Certainty assessment**	15	Describe any methods used to assess certainty (or confidence) in the body of evidence for an outcome.	**Yes**	Section 2.2 (NOS ≥ 7 = high quality; PROBAST risk-of-bias domains)
***RESULTS***				
**Study selection**	16a	Describe the results of the search and selection process, including findings of any searches of other sources to identify studies, from initial number of records identified to final number of studies included, preferably using a flow diagram.	**Yes**	Section 2.1; PRISMA flow diagram
**Study selection**	16b	Cite studies that might appear to meet the inclusion criteria but which were excluded, and explain why they were excluded.	**Yes**	Section 3.1 (exclusion reasons); excluded studies list available on request
**Study characteristics**	17	Cite each included study and present its characteristics.	**Yes**	Section 3.2, Section 3.3, Section 3.4 and Section 3.5; Table 1 (main); Table A3
**Risk of bias in studies**	18	Present assessments of risk of bias for each included study.	**Yes**	Table 4 (quality assessment; NOS and PROBAST ratings)
**Results of individual studies**	19	For all outcomes, present, for each study: (a) summary statistics for each group (where appropriate) and (b) an effect estimate and its precision (e.g., a confidence interval), preferably using structured tables or plots.	**Yes**	Table 2 and Table 3; narrative in Section 3.2, Section 3.3, Section 3.4 and Section 3.5
**Results of syntheses**	20a	For each synthesis, briefly summarize the characteristics and risk of bias among contributing studies.	**Yes**	Section 3.2, Section 3.3, Section 3.4 and Section 3.5; Table 4
**Results of syntheses**	20b	Present results of all statistical syntheses conducted. If meta-analysis was carried out, present for each the summary estimate and its precision and measures of statistical heterogeneity.	**N/A**	Narrative synthesis; no meta-analysis performed
**Results of syntheses**	20c	Present results of all investigations of possible causes of heterogeneity among study results.	**Yes**	Section 4 (Discussion)
**Results of syntheses**	20d	Present results of all sensitivity analyses conducted to assess the robustness of the synthesized results.	**N/A**	Not applicable
**Reporting biases**	21	Present assessments of risk of bias due to missing results (arising from reporting biases) for each synthesis assessed.	**Partial**	Section 4.5 (discussed narratively)
**Certainty of evidence**	22	Present assessments of certainty (or confidence) in the body of evidence for each outcome assessed.	**Yes**	Section 4; Conclusions
***DISCUSSION***				
**Discussion**	23a	Provide a general interpretation of the results in the context of other evidence.	**Yes**	Section 4.1, Section 4.2, Section 4.3, Section 4.4, Section 4.5 and Section 4.6
**Discussion**	23b	Discuss any limitations of the evidence included in the review.	**Yes**	Section 4.5
**Discussion**	23c	Discuss any limitations of the review processes used.	**Yes**	Section 4.5
**Discussion**	23d	Discuss implications of the results for practice, policy, and future research.	**Yes**	Section 4.5 and Section 4.6; Conclusions
***OTHER INFORMATION***				
**Registration and protocol**	24a	Provide registration information for the review, including register name and registration number, or state that the review was not registered.	**Yes**	Section 2.1 (PROSPERO CRD42026XXXXXX—placeholder)
**Registration and protocol**	24b	Indicate where the review protocol can be accessed, or state that a protocol was not prepared.	**Partial**	PROSPERO registration (see above)
**Registration and protocol**	24c	Describe and explain any amendments to information provided at registration or in the protocol.	**N/A**	No amendments
**Support**	25	Describe sources of financial or other support for the review, and the role of the funders or sponsors in the review.	**Yes**	To be completed at submission
**Competing interests**	26	Declare any competing interests of review authors.	**Yes**	To be completed at submission
**Availability of data, code and other materials**	27	Report which of the following are publicly available and where they can be found: template data collection forms; data extracted from included studies; data used for all analyses; analytic code; any other materials used in the review.	**Partial**	Available from corresponding author on reasonable request

Note. Items marked ‘Partial’ indicate that the criterion is addressed but not fully met. Items marked ‘N/A’ are not applicable to the narrative synthesis design of this review. Item numbering follows Page [21].

**Table A2:** Full electronic database search strings.

Database	Full Search String
**PubMed/MEDLINE**	(schizophrenia[MeSH] OR “schizoaffective disorder”[MeSH] OR “psychotic disorder”[MeSH] OR psychosis[tiab] OR schizophrenia[tiab]) AND (stabilized[tiab] OR remission[tiab] OR “maintenance treatment”[tiab] OR outpatient[tiab]) AND (relapse[tiab] OR recurrence[tiab] OR rehospitalization[tiab] OR “symptom exacerbation”[tiab] OR decompensation[tiab]) AND (biomarker[tiab] OR EEG[tiab] OR ERP[tiab] OR “mismatch negativity”[tiab] OR MMN[tiab] OR P300[tiab] OR “event-related potential”[tiab] OR MRI[tiab] OR neuroimaging[tiab] OR “brain volume”[tiab] OR “cortical thickness”[tiab] OR “gray matter”[tiab] OR cytokine[tiab] OR interleukin[tiab] OR “C-reactive protein”[tiab] OR cortisol[tiab] OR prolactin[tiab] OR inflammatory[tiab] OR “gene expression”[tiab] OR proteomic[tiab] OR “digital phenotyping”[tiab] OR smartphone[tiab] OR “passive sensing”[tiab] OR “ecological momentary assessment”[tiab]) AND (“1990”[PDAT]:”2026”[PDAT])
**Scopus**	TITLE-ABS-KEY((schizophrenia OR “schizoaffective disorder” OR “psychotic disorder” OR psychosis) AND (stabilized OR remission OR “maintenance treatment” OR outpatient) AND (relapse OR recurrence OR rehospitalization OR “symptom exacerbation” OR decompensation) AND (biomarker OR EEG OR ERP OR “mismatch negativity” OR MMN OR P300 OR “event-related potential” OR MRI OR neuroimaging OR “brain volume” OR “cortical thickness” OR “gray matter” OR cytokine OR interleukin OR “C-reactive protein” OR cortisol OR prolactin OR inflammatory OR “gene expression” OR proteomic OR “digital phenotyping” OR smartphone OR “passive sensing” OR “ecological momentary assessment”)) AND PUBYEAR > 1989 AND PUBYEAR < 2026
**Web of Science**	TS = ((schizophrenia OR “schizoaffective disorder” OR “psychotic disorder” OR psychosis) AND (stabilized OR remission OR “maintenance treatment” OR outpatient) AND (relapse OR recurrence OR rehospitalization OR “symptom exacerbation” OR decompensation) AND (biomarker OR EEG OR ERP OR “mismatch negativity” OR MMN OR P300 OR “event-related potential” OR MRI OR neuroimaging OR “brain volume” OR “cortical thickness” OR “gray matter” OR cytokine OR interleukin OR “C-reactive protein” OR cortisol OR prolactin OR inflammatory OR “gene expression” OR proteomic OR “digital phenotyping” OR smartphone OR “passive sensing” OR “ecological momentary assessment”)) AND PY = (1990–2026)
**PsycINFO**	(schizophrenia OR schizoaffective OR psychotic OR psychosis) AND (stabilized OR remission OR maintenance OR outpatient) AND (relapse OR recurrence OR rehospitalization OR exacerbation OR decompensation) AND (biomarker OR EEG OR ERP OR “mismatch negativity” OR P300 OR neuroimaging OR MRI OR cytokine OR interleukin OR “C-reactive protein” OR cortisol OR prolactin OR inflammatory OR “gene expression” OR “digital phenotyping” OR smartphone OR “passive sensing” OR “ecological momentary assessment”)—limited to peer-reviewed journals, 1990–2026
**Embase**	(‘schizophrenia’/exp OR ‘schizoaffective disorder’/exp OR ‘psychotic disorder’/exp OR psychosis:ti,ab) AND (stabilized:ti,ab OR remission:ti,ab OR ‘maintenance treatment’:ti,ab OR outpatient:ti,ab) AND (relapse:ti,ab OR recurrence:ti,ab OR rehospitalization:ti,ab OR ‘symptom exacerbation’:ti,ab OR decompensation:ti,ab) AND (biomarker:ti,ab OR EEG:ti,ab OR ‘mismatch negativity’:ti,ab OR MMN:ti,ab OR P300:ti,ab OR MRI:ti,ab OR neuroimaging:ti,ab OR cytokine:ti,ab OR interleukin:ti,ab OR ‘C-reactive protein’:ti,ab OR cortisol:ti,ab OR prolactin:ti,ab OR inflammatory:ti,ab OR ‘gene expression’:ti,ab OR ‘digital phenotyping’:ti,ab OR smartphone:ti,ab OR ‘passive sensing’:ti,ab OR ‘ecological momentary assessment’:ti,ab) AND [1990–2026]/py

Note. Search conducted January 1990–March 2026 across all five databases with no language restrictions. Search strings adapted to database-specific syntax (MeSH terms for PubMed; field codes for Scopus, Web of Science, and Embase; controlled vocabulary for PsycINFO). All database searches were conducted by V.R. and verified by L.R.

**Table A3:** Eligibility criteria (PICOS framework)—Full specification.

PICOS Domain	Criterion	Specification	Decision
**Population (P)**	Diagnosis	Individuals meeting DSM-IV/5 or ICD-10/11 criteria for a schizophrenia-spectrum disorder: schizophrenia, schizoaffective disorder, schizophreniform disorder, or unspecified psychotic disorder	**Include**
**Population (P)**	Clinical status at entry	Clinical stabilization defined by ≥6 months of treatment, absence of acute psychotic episode, or standardized remission criteria	**Include**
**Population (P)**	Age	Any age (adults and adolescents)	**Include**
**Population (P)**	Setting	Outpatient, community, or post-hospitalization follow-up settings	**Include**
**Population (P)**	Treatment-resistant	Studies exclusively examining treatment-resistant populations without a stabilized comparison group	**Exclude**
**Population (P)**	First-episode only (no stabilized follow-up)	Studies exclusively examining first-episode psychosis without follow-up into the stabilized phase	**Exclude**
**Index Test/Predictor (I)**	Neurophysiological	EEG/ERP measures including MMN (duration, frequency, pitch, double-deviant), P300, N100, P50 gating at any assessment timepoint	**Include**
**Index Test/Predictor (I)**	Neuroimaging	Structural MRI (gray matter volume, cortical thickness, subcortical volumes), functional MRI, diffusion tensor imaging, PET	**Include**
**Index Test/Predictor (I)**	Blood-based	Cytokines (IL-6, TNF-α, IL-1β, IFN-γ, IL-10), CRP, cortisol/cortisol awakening response, prolactin, other neuroendocrine markers	**Include**
**Index Test/Predictor (I)**	Molecular/genomic	Peripheral gene expression profiling, polygenic risk score (PRS), telomere length, epigenetic markers	**Include**
**Index Test/Predictor (I)**	Digital phenotyping	Passive smartphone sensing (GPS, accelerometry, screen time, social communication), wearable devices (HRV, activity, sleep), ecological momentary assessment (EMA)	**Include**
**Index Test/Predictor (I)**	Assessment timing	Baseline biomarker assessment (single timepoint) or serial assessments during follow-up	**Include**
**Comparator (C)**	Comparator	Patients who do not relapse during the follow-up period; healthy controls (where used in biomarker validation)	**Include**
**Outcome (O)**	Primary outcome	Psychotic relapse as a primary or secondary outcome, operationalized through: hospitalization records, structured clinical interview thresholds (e.g., PANSS increase ≥25% or score ≥4 on P1/P2/P3), or expert consensus using standardized criteria	**Include**
**Outcome (O)**	Secondary outcomes	Time to relapse, number of relapses, treatment response (partial outcome), functional deterioration as relapse proxy	**Include**
**Outcome (O)**	Outcome not assessed	Studies that do not report relapse or a relapse-equivalent outcome (e.g., purely cross-sectional biomarker studies with no prospective follow-up)	**Exclude**
**Study Design (S)**	Design—include	Longitudinal prospective cohort studies with biomarker assessment at baseline or serially and prospective relapse outcome ascertainment	**Include**
**Study Design (S)**	Follow-up duration	Minimum 6 months of prospective follow-up after baseline biomarker assessment	**Include**
**Study Design (S)**	Case-control	Case–control studies without prospective relapse outcome prediction (i.e., retrospective biomarker comparison between relapsers and non-relapsers ascertained post hoc)	**Exclude**
**Study Design (S)**	Conference abstracts	Conference abstracts, letters, editorials, and narrative reviews without peer-reviewed empirical data	**Exclude**
**Study Design (S)**	Language	No language restriction; all peer-reviewed publications included regardless of language	**Include**
**Study Design (S)**	Publication date	January 1990 through March 2026	**Include**

Note. PICOS = Population, Index test/predictor, Comparator, Outcome, Study design. Decision cells indicate whether the criterion was used to include or exclude studies. Criteria applied independently by two reviewers (V.R., L.R.); disagreements resolved by discussion or a senior reviewer (G.Ma.).

**Table A4:** Risk of bias summary figure. Color-coded assessment of methodological quality for each included study across six domains. Green = low risk/high quality; yellow = moderate risk; red = high risk/low quality; grey = not applicable. Ratings are consistent with the Newcastle-Ottawa Scale and PROBAST assessments reported in Table 1.

	Low Risk/High Quality		Moderate Risk		High Risk/Low Quality		Not Applicable
Study	Participant Selection	Biomarker Assessment	Outcome Definition	APD Confounding	Adherence Control	Validation	Overall
Bodatsch [19]							
Hamilton [23]							
Nakajima [24]							
Hamilton [25]							
Giordano [26]							
Light & Braff [27]							
Higashima [28]							
Kim [29]							
De Wilde [30]							
Van Tricht [31]							
Duncan [32]							
Brockhaus-Dumke [33]							
Khandaker [34]							
Stojanovic [35]							
Mondelli [36]							
Gassó [37]							
Pawelczyk [38]							
Landi [39]							
De Nijs [40]							
Adler [41]							
Garyfalli [42]							

Note. Green = low risk/high quality; Yellow = moderate risk; Red = high risk/low quality; Grey = not applicable. APD = antipsychotic drug. Ratings consistent with the Newcastle-Ottawa Scale and PROBAST assessments in Table 1.
